# A Computational Model of Immanent Accent Salience in Tonal Music

**DOI:** 10.3389/fpsyg.2019.00317

**Published:** 2019-03-29

**Authors:** Erica Bisesi, Anders Friberg, Richard Parncutt

**Affiliations:** ^1^Centre for Systematic Musicology, University of Graz, Graz, Austria; ^2^Laboratory “Perception and Memory”, Department of Neuroscience, Institut Pasteur, Paris, France; ^3^Department of Speech, Music and Hearing, School of Electrical Engineering and Computer Science, KTH Royal Institute of Technology, Stockholm, Sweden

**Keywords:** immanent accents, salience, music expression, music analysis, computational modeling

## Abstract

Accents are local musical events that attract the attention of the listener, and can be either immanent (evident from the score) or performed (added by the performer). Immanent accents involve temporal grouping (phrasing), meter, melody, and harmony; performed accents involve changes in timing, dynamics, articulation, and timbre. In the past, grouping, metrical and melodic accents were investigated in the context of expressive music performance. We present a novel computational model of immanent accent salience in tonal music that automatically predicts the positions and saliences of metrical, melodic and harmonic accents. The model extends previous research by improving on preliminary formulations of metrical and melodic accents and introducing a new model for harmonic accents that combines harmonic dissonance and harmonic surprise. In an analysis-by-synthesis approach, model predictions were compared with data from two experiments, respectively involving 239 sonorities and 638 sonorities, and 16 musicians and 5 experts in music theory. Average pair-wise correlations between raters were lower for metrical (0.27) and melodic accents (0.37) than for harmonic accents (0.49). In both experiments, when combining all the raters into a single measure expressing their consensus, correlations between ratings and model predictions ranged from 0.43 to 0.62. When different accent categories of accents were combined together, correlations were higher than for separate categories (*r* = 0.66). This suggests that raters might use strategies different from individual metrical, melodic or harmonic accent models to mark the musical events.

## Introduction

An important aspect of structure in classical Western music is the *accent*: a local event that attracts a listener's attention. This concept of accent is shared by several authors (e.g., Thomassen, 1982; Lerdahl and Jackendoff, 1983; Jones, 1987; Parncutt, 1987, 2003; Drake and Palmer, 1993; Huron and Royal, 1996; Pfordresher, 2003; Müllensiefen et al., 2009; Ammirante and Thompson, 2010; Bisesi and Parncutt, 2011; Parncutt et al., 2013; Friberg and Bisesi, 2014), and is broader than the everyday meaning of simply playing a note louder. Accents can be either evident from the score (immanent) or added by the performer (performed) (Parncutt, 2003). In both cases, the perceptual salience of an accent may be defined as its perceptual importance, or the degree to which it attracts a listener's attention. In this study, we focus on immanent accents. They are associated with grouping (phrasing), meter, melody and harmony.

- *Grouping accents* occur at the beginning and end of note groups, at any different hierarchical level of phrasing (Drake and Palmer, 1993), and may similarly be related to other formulations of the grouping structure (cf. Deutsch, 1982; Lerdahl and Jackendoff, 1983).
- *Metrical accents* are based on a similar hierarchical cognitive structure—the hypermeter—which consists of a tactus (or main beat), slower pulses (perceived or implied pulse trains or metrical levels containing several tactus events), and faster pulses into which the tactus is subdivided (Cooper and Meyer, 1960; Lerdahl and Jackendoff, 1983; Palmer and Krumhansl, 1990; Handel, 1998). In a first approximation, we defined the salience of a metrical accent as the number of different levels of pulsation to which it belongs. In a better approximation, we considered also the dependence of pulse salience on tempo: pulses closer to about 100 events per minute or 600 ms tend to be more salient (Yeston, 1976; Handel and Oshinsky, 1981; Beauvillain and Fraisse, 1984). We then added the salience of the pulses to which each event belongs; for example, the note at the start of a measure is part of both the measure-pulse and the beat-pulse. When applying this to the score, we assumed that notated meter corresponds to the perceived meter.
- *Melodic accents* consist of turns (peaks and valleys of the melodic contour), skips (leaps or disjoint intervals between consecutive tones, with the accent falling on the second tone of the skip) or both. Melodic accents appear to play an important role in the tonal Western repertoire. Expressive delaying and lengthening is common in the vicinity of melodic peaks, and to a lesser extent near melodic valleys; this effect is clearly audible on commercial CD recordings of the music of well-known composers. Huron and Royal (1996) listed and evaluated different theories of melodic accent, all of which may be considered to be variants on the following two principles. First, the local maxima and minima in a melody (registral extremes, pivot points) are likely to be heard as accented; the higher a maximum or the lower a minimum, the greater the accent. Second, tones following leaps are likely to be heard as accented; the bigger the interval, the bigger the accent. In addition, different authors have suggested that high tones in a melody are more accented than low tones, and tones following rising leaps are more accented than tones following falling leaps. For example, Thomassen (1982) developed a model of melodic accent based on three-tone segments of melodic contours, or sequences of two melodic intervals. In his model, predicted accent saliences depend only on whether the contour rises or falls, or the tone is repeated; they do not directly depend on interval size or the relative pitch of peaks or valleys. Huron and Royal compared various models of accent and found some support for Thomassen's model: “Melodic accent may be a relatively weak factor in rhythmic perception and musical organization” (p. 509). However, they did not test combinations of different simpler models. Inspired by these anecdotal evidences, we developed a theoretical and algorithmic approach to melodic accents that innovatively combines the intuitions of previous authors.
- *Harmonic accents* may occur at events with change of harmonic tension like harmonic climaxes or resolutions, and include harmonic dissonances (sonorities that include dissonant intervals) and harmonic changes (surprising chords and modulations or root changes) (Lerdahl and Jackendoff, 1983; Dawe et al., 1993). Harmonic accents in Western tonal music can be divided into two main categories, simultaneous and successive, which we call vertical and horizontal by allusion to vertical and horizontal structures in conventional musical scores:

- *Vertical harmonic accents* are related to (or caused by) the vertical (or simultaneous) dissonance of individual sonorities (cf. Smith and Cuddy, 1989). Previous attempts to model harmonic dissonance in terms of roughness and harmonicity directly from the spectrum (Hutchinson and Knopoff, 1978; Aures, 1985; Parncutt, 1988, 1989; Bigand et al., 1996) had mixed success; currently, we know of no model that plausibly predicts the perceived dissonance of any sonority in the chromatic scale. For example, no such model can explain why the diminished triad is generally considered more consonant than the augmented. For this reason, we decided to model vertical dissonance in a quite different way. We assumed that consonance depends primarily on familiarity (Cazden, 1945; Parncutt and Hair, 2011); familiarity depends in turn on the number of times a sonority happens in the music to which a listener has been exposed.
- *Horizontal harmonic accents* are associated with temporal relationships between successive sonorities. They are a measure of the horizontal (successive) change of tension or surprise that we experience when a sonority sounds noticeably different from preceding sonorities (cf. Dawe et al., 1994). Our model is inspired by a simple octave-generalized formulation of harmonic pitch-pattern recognition in virtual pitch perception (Terhardt et al., 1982; Parncutt, 1988). According to Parncutt (1988), the predicted pitch-class salience profile for each chord may include pitches at missing fundamentals that are not notated in the score (e.g., the tone G in the diminished triad BDF); Parncutt (1988) also distinguished between more or less salient tones (e.g., in the chord CEG the tone C is more salient than E or G). Thus, the pitch-class salience profile is a perceptual representation of each chord that is different from both the acoustic representation (spectrum) and the notational representation (notes), because it includes missing fundamentals and variations in pitch salience. The Parncutt (1988) model was tested by Parncutt (1989, 1993) and appears to work well-enough for our purposes. In our model, we assumed that horizontal harmonic accents are essentially the inverse of pitch commonality as defined by Parncutt (1989). The computation is inspired by Krumhansl's (1990) use of correlation coefficients to compare the tone profiles of successive sonorities. The resulting pitch-class salience profile is a vector with salience values for the 12 pitches in the chromatic scale. We have been interested in modeling the harmonic accent of a current sonority that follows a series of preceding sonorities. That is essentially the same as evaluating the degree to which an event is “harmonically surprising.” Suitable foundations for modeling harmonic surprise can be also found in Huron and Parncutt (1993), Bigand and Parncutt (1999), and Sapp (2011).

Patterns of tone or event salience in music depend on general psychological principles that determine object or event salience in vision and other perceptual modalities. Consider first harmonic accent. A visual element is salient if it contrasts with its background or with surrounding elements in some way (color, motion, orientation, form, luminance; Turatto and Galfano, 2000). Similarly, harmonic accents are perceived at chords that differ markedly from preceding chords in consonance or tonality. Regarding melodic accent, a visual event becomes more salient if it moves suddenly (Abrams and Christ, 2003). In the physical world, that may be because the velocity of the source changes due to an external force, which is ecologically interesting for the perceiving organism. Similarly, attention is drawn to an easily audible melodic tone that suddenly moves (a melodic leap) or changes direction (a local peak of the melodic contour). Metrical accents attract attention for a different reason: they permit the organism to predict future events (Jones and Boltz, 1989). This principle may also apply to serial grouping accents at the starts of musical phrases, which are analogous to the starts of speech phrases. In both cases, salience is determined in part by temporal primacy and recency (Gupta et al., 2005). Metrical and grouping accents may also be byproducts of the parsing of continuous perceptual streams into hierarchically structured events. This process involves perceptual grouping or chunking to reduce the cognitive load (Kurby and Zacks, 2008).

Drake and Palmer (1993) investigated grouping (which they called rhythmic grouping), metrical accent, and melodic accent. Assuming that groups begin and end with accents, and that accents are defined as events that attract attention for any reason, we could say that Drake and Palmer investigated grouping, metrical, and melodic accents, and how pianists emphasize them. They found that all three kinds of immanent accent can be associated with increases in loudness, slowing of local tempo (delaying or lengthening), and/or increases in the degree of legato overlap (articulation). Performed accents were strongest at grouping accents and weakest at metrical accents, but grouping accents only affected performance at the ends of groups—not at the starts. The psychological function of accent structures, according to Drake and Palmer, is to provide cues to segmentation. Accent positions are determined by Gestalt principles of proximity and similarity. Consequently, expression in music performance may have the function of clarifying segmentation to the listener, which facilitates the intuitive understanding of musical structure. “Performers use systematic variations to highlight important aspects of musical structure, which may facilitate listeners' segmentation of music into units of a size that can be analyzed” (p. 376). On the whole, the results of Drake and Palmer were consistent with the simple linear addition of effects of different accents. However, there was also evidence for interactions between accents: “performance variations related to melodic accent structure were affected by the presence of absence or other accent structures” (p. 375).

Melodic and harmonic accents are also related to the three primary factors of melodic expectation as identified by Margulis (2005): tonal stability, melodic proximity, and melodic direction. In this paper, we will concentrate on metrical, melodic and harmonic accents. Different from Margulis (2005), we will model harmonic and melodic tension separately, and then address the question of their relationship empirically (i.e., by examining the data).

All categories of immanent accent introduced so far can be involved when a performer introduces expression into performance. A performer may do something special to an accented tone or sonority to “bring it out”—to attract further attention to it (Clarke, 1988, 1993). In other words, expressive performance may involve a further enhancement and strenghtening of immanent accents by reinforcing them so that they become performed accents. For instance, in piano music performed accents involve changes in timing, dynamics, and articulation; they vary in amplitude, form, and duration (Kurkela, 1995). A piano performer may slow the local tempo or (equivalently) add extra time in the vicinity of certain kinds of immanent accent, or change dynamics (usually get louder) or articulation (usually get more legato or increase overlap between successive tones), in consistent ways (Sundberg et al., 1983; Drake and Palmer, 1993). This relationship is complex and may depend on many factors, such as musical and personal style, local and cultural context, intended emotion or meaning, and acoustical and technical constraints. All these aspects can serve as input for a new model of expressive performance. An example is provided in Caron et al. (2019), who classified several harpsichord performances by means of local variations of tempo in the vicinity of immanent accents.

A review on the several approaches to modeling music structure (including the way to interpret it in expressive performance) was provided by Bisesi and Windsor (2016). A first possibility—which our approach is based on—is the grammatical approach, whereby a computer reads the score and then uses a set of parameters and rules to predict immanent structures or, in the case of music performance, to translate these structures into local deviations of tempo, dynamics, articulation, and timbre (Sundberg et al., 1983; Friberg et al., 2006). This corresponds to a top-down approach in the sense that a set of principles is assumed from which mathematical rules are formulated, then experimental data are collected with the purpose of assessing how much the participants' answers agree with the original principles. A second (bottom-up) approach usually involves machine learning: it is based on the statistical analysis of a database of human responses (involving either music perception or expressive performances), which is used to train a machine learning system to predict collected data (Widmer and Tobudic, 2003; Widmer and Goebl, 2004; Cancino-Chacón, 2018). A similar bottom-up machine learning approach was used by Müllensiefen et al. (2009) to model perceived accent (immanent and performed) in pop melodies starting from a rather large set of melodic and rhythmic features extracted from the score. This approach was also used to investigate the perceived immanent accents in 60 melodies of Western art music (Friberg et al., submitted^1^). The grammatical approach has the advantage that expressive strategies can be understood in music-theoretical terms, which makes it more suitable for music analytical and pedagogical work. The machine-learning approach has the advantage that it is more closely linked to real performances or performance traditions in all their details, and offers the possibility of convincingly imitating the style of a given performer without distorting it in the direction of a given prior theory.

We already pointed out the role of accentuation by formulating a preliminary computational model of metrical and melodic contour accents (Bisesi and Parncutt, 2011; Parncutt et al., 2013). Our approach was defined from a top-down theoretical perspective according to previous research (Parncutt, 2003). By adopting intuitive principles for selection of important events as outlined in Parncutt (2003), we estimated their relative degree of importance, or *salience* (a peak centered on the accented notes), range of action (number of notes involved before and after the peak), and slope (smoothness or steepness of the profile moving toward and departing from the peak). This formulation also extended Director Musices (DM)—a performance rendering system that introduces expressive variations into input score files according to the musical context (Sundberg et al., 1983; Friberg et al., 2000, 2006)^2^—in a new direction, by relating the expressive features of a performance not only to global or intermediate structural properties (i.e., different levels of phrasing), but also to local events (individual notes corresponding to accents) in a systematic way. In a subsequent study, our preliminary formulation of the accent model was applied to different musical styles (Friberg and Bisesi, 2014).

Despite providing interesting results from the viewpoint of music analysis and potential insight in the field of expressive performance, all our previous studies suffered the limitation of being exclusively theoretical and requiring a number of manual adjustments depending on any specific piece. In the current study, we present a novel computational model of immanent accent salience in tonal music, which extends and improves previous research in the following ways. First, we are now focusing on three—instead of two—different types of immanent accent, which are modeled and evaluated both separately and in combination: besides meter and melodic contour, we included also vertical and horizontal aspects of harmony. Second, our previous models for metrical and melodic accents (Friberg and Bisesi, 2014,^3^) have been improved by means of a refinement in the parameter space and a rescaling in the case of metrical accents, and a complete reformulation in the case of melodic accents. Third, predictions for all categories have been compared with data from two different experiments, and corresponding algorithms optimized. Finally, for the first time in the context of accent theory, the model is totally automatic.

As before, positions and saliences for metric, melodic and harmonic accents are predicted by adopting a music theoretic perspective; the extent to which these concepts are activated through listening is the focus of a separate study (Friberg et al., submitted)^1^. The method adopted to develop the new accent model was analysis by synthesis. We started with a set of prescriptions combining and extending existing formulations, and then compared predictions with accents marked by musicians and expert music theorists on the scores of ten piano pieces differing in musical structure and style. As we did not include aspects related to instrumental timbre, the choice to restrict our study to the solo piano repertoire was arbitrary and dictated only by consistency.

The structure of the paper is as follows. In section Methods, we describe the methodology for the empirical studies and rule development. We then present the model, in section The Accent Model. Section Results provides an overview of the results, and section Conclusions and Discussion contains the main conclusions followed by a discussion.

## Methods

### Experiments

In order to collect data on musicians' intuitions about immanent accents, two experiments were conducted. In the first experiment, we focused on only two pieces and asked participants to mark melodic and harmonic accents on the score. We were also interested in comparing and relating the Phrase arch rule in Director Musices (based on phrase analysis: Friberg, 1995) with the current approach (based on accent theory: Parncutt, 2003; Bisesi and Parncutt, 2011; Parncutt et al., 2013; Friberg and Bisesi, 2014), and therefore asked participants to mark also phrase boundaries and climaxes. Following a preliminary phrase analysis (not reported in this paper), as well as suggestions provided by most of the raters in a debriefing, we decided to carry out a second experiment including also metrical accents. We improved the quality of the experimental design in three ways: by increasing the number of pieces from two to ten (increasing both the number of marked musical events and the number of musical structures and styles), by raising the level of expertise of the raters (involving expert music theorists instead of mostly students), and by exploring their strategies in an interview.

### Experiment 1

#### Participants

Sixteen musicians participated in the study (12 males and 4 females). Fourteen were students recruited from the University of Music and Performing Arts in Graz (9 students in music theory), the Karl-Franzens University in Graz (3 students in musicology), and Italian Music Conservatories (2 students in music theory); the other 2 participants were musicologists. All participants were also performers.

#### Materials

The scores of two piano pieces were used, consisting of 239 sonorities in total, where a new sonority is defined every time there is an onset in any voice. The pieces were two Chopin Preludes with different phrasing, metrical, melodic and harmonic structures^4^—the Prelude Op. 28 No. 6 in B minor and the Prelude Op. 28 No. 7 in A major. The scores were created by means of the free music composition and notation software MuseScore 2.1^5^. All the notated agogical and dynamical indications and all of the phrase marks were removed.

#### Procedure

Participants received the musical scores on paper, one sheet of A4 for each, and marked the melodic and harmonic accents in writing using a coding scheme where selected melodic-contour and harmonic accents were indicated with C_x_ and H_x_, respectively, x being the relative importance (or *salience*) of the accent on a scale from 1 to 5. Participants in the study were free to mark any tone in the score as an accent and then evaluate its strength on the 5-point scale. In other words, any “sonority” (as defined above) could be marked. In addition, they made a hierarchical analysis of the phrase structure by marking the boundaries (start and end) and climaxes of each phrase and subphrase; they also indicated the hierarchical level of each boundary and climax. The phrase analysis data were not used in the present paper. Participants did not listen to any sound examples. Before doing the task, they were asked to read a set of guidelines, where the concept of musical accent as a note or chord that catches the listeners' attention and the different theoretical principles adopted in our formulation were presented, together with a musical example^6^ showing one possible way of marking the accents. In the guidelines, we clarified that these examples were not a strict prescription and asked the participants to follow their musical intuition. The example in the guidelines was different from the pieces to be used in the study and had been analyzed by the three authors, all with extensive piano performing experience [Note that, although the three authors agreed on the approach, there were also interesting differences between them in approaching to the task. One author (Parncutt) read the scores and imagined the sound, while the others (Bisesi and Friberg) listened to performances; of those, one (Bisesi) had performed several pieces of the repertoire being analyzed, while the other (Friberg) usually performed a different repertoire (jazz)]. After the main task, participants were asked to fill a questionnaire to provide feedback about any difficulties they had encountered and their opinions about the methodology.

### Experiment 2

#### Participants

Five male music theorists participated in the experiment, with different theoretical backgrounds and approaches to music analysis in their own teaching and research. They were respectively: two professors of harmony and/or music theory—one originally from North America, following a traditional approach to harmony, and also expert in the Baroque-style repertoire; another originally from Germany, who generally prefers a perception- and performance-informed analytical approach, and with high experience in post-tonal music; the other three participants, all Italian, were a Schenkerian-oriented musicologist, a neo-Riemannian-oriented analyzer, and a musicologist specialized in analysis and perception of music. All participants were also composers and/or expert performers. The motivation for recruiting participants with different methodological preferences was to avoid any bias toward specific analytic strategies. Given the small number of participants, we limited ourselves to reporting some of the observations they provided (cf. section Raters' Comments), without trying to establish any formal relationship between our model and other approaches to music analysis.

#### Materials

We used the scores of 10 different piano pieces belonging to the Classical, Romantic and post-Romantic piano repertoire including part of the material used in Experiment 1. The pieces were chosen for their diversity regarding metrical, rhythmical, melodic and harmonic structure, as well as notated tempo (see Table 1). They consisted of 638 sonorities in total. We included pieces with regular and non-regular metric patterns, slow and fast marked tempi, low and high note densities, short- and long-range melodies, small and big melodic intervals, low and high amount of dissonance, less or more tonal ambiguity. Although the amount of music was relatively small and limited to few composers for piano, the sample was reasonably diverse with respect to note density, rhythmic figuration, register, melodic and motivic patterns, passing and neighbor tones/chords, dissonances and harmonic progressions, so as to allow a systematic examination of the relationship between textural properties of Western tonal music and the above-named categories of accents. Small datasets also permit closer inspection of the data collected (see sections Overview of Accent Marks and A Qualitative Comparison Between Models and Data and **Figures 3–5** below). The scores were created by means of the free music composition and notation software MuseScore 2.1. All the notated agogic and dynamic indications and all phrase marks had been removed.

**Table 1:** Excepts analyzed by music theorists in Study 2 (including part of the material used in Study 1).

	**Excerpt**	**Main musical aspects**
1	W. A. Mozart—Sonata K. 533, 2nd Mvt. (bar 1–10)	Classical style, hybrid thematic structure^a^ (antecedent + consequent with extension), slow tempo, non-motoric rhythmic pattern, 3/4 meter, melodic line in the upper voice, big leaps, passing tones and grace notes, standard harmonic progression.
2	W. A. Mozart—Sonata K. 576, 2nd Mvt. (bar 1–8)	Classical style, thematic structure of period, slow tempo, non-motoric rhythmic pattern, 3/4 meter, melodic line in the upper voice, intermediate leaps, passing tones and grace notes, standard harmonic progression.
3	L. van Beethoven—Variations WoO 70, Theme (bar 1–8)	Classical style, hybrid period structure (antecedent + continuation → cadential), intermediate tempo, motoric rhythmic pattern, 6/8 meter, melodic line in the upper voice, big leaps, standard harmonic progression.
4	L. van Beethoven—Variations WoO 70, Var. I (bar 1–8)	Classical style, hybrid period structure (antecedent + continuation → cadential), fast tempo, motoric rhythmic pattern, 6/8 meter, mixed metric structure, melodic line in the upper voice, small leaps, passing tones, standard harmonic progression.
5	L. van Beethoven—Variations WoO 70, Var. III (bar 1–8)	Classical style, hybrid period structure (antecedent + continuation → cadential), fast tempo, motoric rhythmic pattern, 6/8 meter, overlap of metric and melodic accents, melodic line shared between voices, big leaps, standard harmonic progression.
6	F. Chopin-Prelude Op. 28 No. 6 (bar 1–8) (in Study 1, complete)	Romantic style, sentence structure, slow tempo, non-regular rhythmic pattern, 3/4 meter, melodic line shared between voices, big leaps, modulations, accidentals.
7	F. Chopin—Etude Op. 25 No. 7 (bar 1–9)	Romantic style, free formal structure, slow tempo, mixed rhythmic pattern, 3/4 meter, melodic line shared between voices, big leaps, modulations, accidentals.
8	F. Chopin—Prelude Op. 28 No. 7 (complete)	Romantic style, compound period structure, intermediate tempo, rhythmic regularity along the piece but not inside bars, 3/4 meter, melodic line in the upper voice, big leaps, standard harmonic progression, accidentals.
9	F. Schubert / F. Liszt—Der Doppelgänger, piano transcription (bar 43–63)	Romantic style (not conventional), free formal structure, slow tempo, non-regular rhythmic pattern, 3/4 meter, melodic line in the inner voice, non-standard harmonic progression, non-conventional modulations (according to the Classical-Romantic harmony), accidentals.
10	R. Wagner—Elegie WWV 93 (complete)	Post-Romantic style, free formal structure (basic compound idea + interrupted continuation), slow tempo, non-regular rhythmic pattern, 3/4 meter, melodic line in the upper voice, big leaps, non-standard harmonic progression, non-conventional modulations (according to the Classical-Romantic harmony), accidentals.

a *Formal analysis performed according to Caplin (2013)*.

#### Procedure

The task was similar to that of the previous experiment, with the difference that phrasing analysis was no longer included as a task, and the categories of accent were extended to include metrical/rhythmic accents (marked as M_x_, cf. previous section Procedure). The guidelines were adapted accordingly. As in the previous case, the principles presented in the guidelines were not strict and participants were encouraged to follow their own musical intuition. Each piece was marked with a tempo indication, but there were no specific instructions on how to approach the task—whether by listening, playing or imagining the music. After the main task, participants were asked in an interview to comment on their strategies for marking the accents in relation to mainstream approaches to music analysis.

### Methodology for Rule Development

Our modeling approach is based on aspects of generative grammar (Lerdahl and Jackendoff, 1983), as well as on the rule-based approach of Sundberg (1988) and Friberg (1991). Like them, we started with the score. The pieces examined were the same used in the experiments; as described above, they were systematically selected to provide contrasting examples of accentuation, as well as of different stylistic conventions. The model was revised several times in an analysis-by-synthesis approach (Friberg et al., 2014). We started with theoretical principles from previous research (Bisesi and Parncutt, 2011; Parncutt et al., 2013; Friberg and Bisesi, 2014), and then adjusted our previous formulation (or formulated new rules) by comparing model predictions with data from the experimental studies. In addition, suggestions provided by the participants during interviews were considered. Given the high variability in participants' ratings, model optimization was done first qualitatively, by focusing on the events that were marked by more raters and/or received the highest ratings. While the first two/three pieces required several adjustments, after the fourth piece the model converged to a stable solution accomplishing leading principles of music theory (hypermeter, melodic climax, harmonic tension, and resolution). A quantitative evaluation on the agreement between raters and model was then carried out.

## The Accent Model

**Figure 1:**
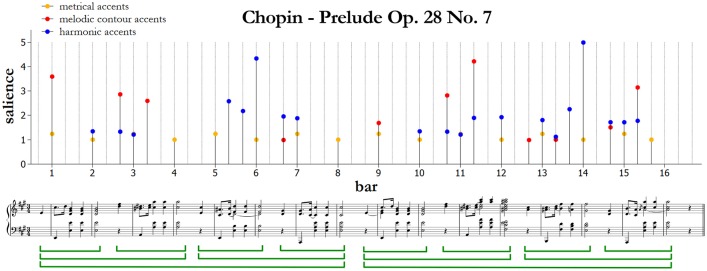
Algorithmic predictions for metrical, melodic, and harmonic accents in Prelude Op. 28 No. 7 by Fryderyk Chopin. An intuitive phrase segmentation is indicated below the score.

We now describe an algorithmic approach to the automatic analysis of musical score that is based on accents, as obtained by analysis by synthesis starting from the above-described experiments. Our model predicts the positions and saliences (i.e., a measure of the degree of importance, from 1 to 5) of metrical, melodic, and harmonic accents based on the music notation. An example of the resulting predicted accents is provided in Figure 1 at the end of the next subsection. In the figure, circles are algorithmic predictions for metrical, melodic and harmonic accents, respectively. Comments to Figure 1 are provided separately in each subsection, concerning different typologies of accents.

### Metrical Accent Model

Our algorithm to predict the positions and saliences of metrical accents has two separate stages. We first mark four different pulses (metrical levels), as detailed in Table 2^7^. Note that the beat corresponds to Level 1. Each note in the score that starts at one of the four subdivisions of the measure according to the table is marked with the corresponding metrical level. For example, a note starting at the onset of the measure is marked with all four metrical levels. At this point each salience value is set to constant value *k* for all metrical levels.

**Table 2:** Notated durations of pulses implied by a given time signature, as used in the algorithm.

	**Metrical level**			
**Time signature**	**Level 0**	**Level 1 (beat)**	**Level 2**	**Level 3**
2/2	1/4	1/2	2/2	4/2
4/2	1/4	1/2	2/2	4/2
2/4	1/8	1/4	2/4	4/4
3/4	1/8	1/4	3/4	6/4
4/4	1/8	1/4	2/4	4/4
3/8	1/8	3/8	6/8	12/8
6/8	1/8	3/8	6/8	12/8
9/8	1/8	3/8	9/8	18/8
12/8	1/8	3/8	6/8	12/8

*If the time signature is 2/2 (with 2 half-notes per measure), for example (top row), Level 0 corresponds to a quarter-note pulse (i.e., 4 beats per measure) and Level 3 corresponds to two measures (4 half notes, or one beat every second measure)*.

In stage 2, the salience values *k* are scaled assuming that the perceptual salience of each pulse depends on the pulse period *P* according to a Gaussian function (Parncutt, 1994). Pulse salience *PS* is computed by this formula:

(1) $$\begin{array}{l} PS_{i}=k\;*\;e^{-0.5\;*\;{\left(\frac{\text{log}P_{i}-\text{log}M}{\text{log}S}\right)}^{2}}\; \end{array}$$

Here, *P*_*i*_ is the pulse period (in seconds) of metrical level *i*. The parameter *M* is a time period (the inverse of tempo) corresponding to the mean of an assumed Gaussian function of pulse salience against the logarithm of pulse period. The parameter *S* is the standard deviation of this distribution. Tentatively, the values are set to *M* = 2 s and *S* = 1.65. These values were determined by trial and error in order to obtain an intuitive and plausible salience hierarchy for different meters and tempi^8^. Finally, the metrical accent salience of each note is computed as the sum of the saliences of all metrical levels that are marked on such note. As a matter of clarity, we remove all metrical accents with a salience *PS* smaller than 1.

Figure 1 shows an example of how metrical accents are computed by the model. Note that the metrical accents simply reflect the notated three-quarter meter; quarter notes are grouped into threes making a three-quarter-note pulse at the barline. There are two adjacent levels of a metrical hierarchy related to the hypermetrical two-bar structure. In the figure, the first beat in each measure has a stronger metrical accent; the other two beats have weaker metrical accents and are not indicated, because a cut-off for metrical accents smaller than 1 has been applied. If no event occurs on a beat, no accent is marked.

### Melodic Contour Accent Model

In our algorithm, we assume that two main factors contribute to the salience of a melodic accent: the size of the leap preceding the accent, and the distance of the accent from the center of the melody's range or ambitus, averaged over the past several notes. We assume that these two factors are multiplied, because if either is zero, musical intuition suggests that the accent will be zero. Thus, we assume that melodic accent salience is proportional to the product of two terms, one (*CS*_1_) depending on the distance of the pitch from the mean pitch and the other (*CS*_2_) depending on the size of the immediately preceding melodic interval.

The salience for the melodic contour accent algorithm is computed in the following way. The running mean pitch for each note is calculated as the mean pitch of all notes starting at the barline two measures before the current note. If there are <10 notes within this range, the average is extended backwards up to the last 10 notes. Let *I*_1_ be the pitch difference to the running mean pitch in semitones, the corresponding contribution to contour salience for each note is defined as

(2) $$\begin{array}{l} CS_{1}=I_{1\;}\;for\;I_{1}>0\\ CS_{1}=0.7\;*\;I_{1}\;for\;I_{1}<0 \end{array}$$

Let *I*_2_ be the pitch interval preceding the current note in semitones. Then the corresponding contribution to melodic contour salience from the preceding interval is defined as

(3) $$\begin{array}{l} CS_{2}=I_{2}\;for\;I_{2}>0\\ CS_{2}=0.2*I_{2}\;for\;I_{2}<0 \end{array}$$

Multiplicative coefficients in *CS*_1_ and *CS*_2_ have been set by trial and error. The combined melodic contour salience is defined by as

(4) $$\begin{array}{l} CS=\frac{\sqrt{CS_{1}*CS_{2}}}{N} \end{array}$$

where *N* is a normalization factor currently set to *N* = 2.5 (by trial and error approach). The calculated melodic salience *CS* is added to the current note provided that it is > 0. Then, the melodic accent is removed on the middle note of three in ascending or descending step-wise motion, and only the highest value of *CS* within the context of three notes is retained. As a matter of clarity, we remove all melodic accents with a salience *CS* smaller than 1. Finally, we apply a saturation rule assigning the value of 5 to all melodic accents higher than 5 (in the same way as for the metrical and harmonic accents). However, these accents are pretty rare.

Figure 1 shows an example of how the salience *CS* for melodic accents is computed by the model. The first melodic accent is at the start of measure 1 (C♯5). It reflects the size of the rising leap (M6) that precedes and attracts attention to it. The accent at the end of measure 2 (F♯5) is more salient than the accent on beat 2 of measure 3 (A5), because the leap preceding the A5 is smaller (a perfect fourth, P4) than the leap preceding the F♯5 (P5). The accent at measure 6 beat 3 corresponds to a valley in the melodic contour. Measure 9 simply repeats measure 1: its accent is smaller than in measure 1 because it's shared among three different sonorities and then the distance from the mean pitch is consequently smaller. The accent on beat 2 of measure 11 is more salient than the analogous accent in measure 3, because the pitch is higher (C♯6). This accent corresponds also to the melodic climax of the piece.

### Harmonic Accent Model

#### Vertical Harmonic Accents

Our model of vertical harmonic accents starts by coding consecutive sonorities as pitch-class-sets (pcs: a numerical representation consisting of groups of unordered pitch classes; Forte, 1973). Our analysis is currently limited to T_*n*_ types^9^ pitch-class-sets in which the two intervallic inversions of non-symmetrical pitch-class-sets are distinguished (Rahn, 1980). For example, major and minor triads belong to the same pitch-class set, but different T_*n*_ types. Then we count how often all possible T_*n*_ sets occur in a large database of Western tonal piano music consisting of the complete J. S. Bach's *Well-Tempered Clavier*, a selection from *Sonatas* by Domenico Scarlatti, all Beethoven piano *Sonatas* and *Variations*, all Chopin *Mazurkas* and *Preludes*, and a selection of pieces by Schumann and Brahms, as both unprepared and prepared sonorities (i.e., sonorities in which the tone onsets are either simultaneous or non-simultaneous). All the pieces have been downloaded from the *KernScores* database,^10^ and then analyzed by means of the *Humdrum Toolkit* (Huron, 1995). The musical score is in *kern* format, and the T_*n*_ type at each note event is calculated by means of the Humdrum algorithm *tntype*. With the purpose of a completely automatized model, all scores are cut into chord slices without any reduction (i.e., ornamental notes were included). In total, our database comprises 346 pieces for a total of 269,547 events (i.e., consecutive sonorities consisting of single notes or chords).

**Figure 2:**
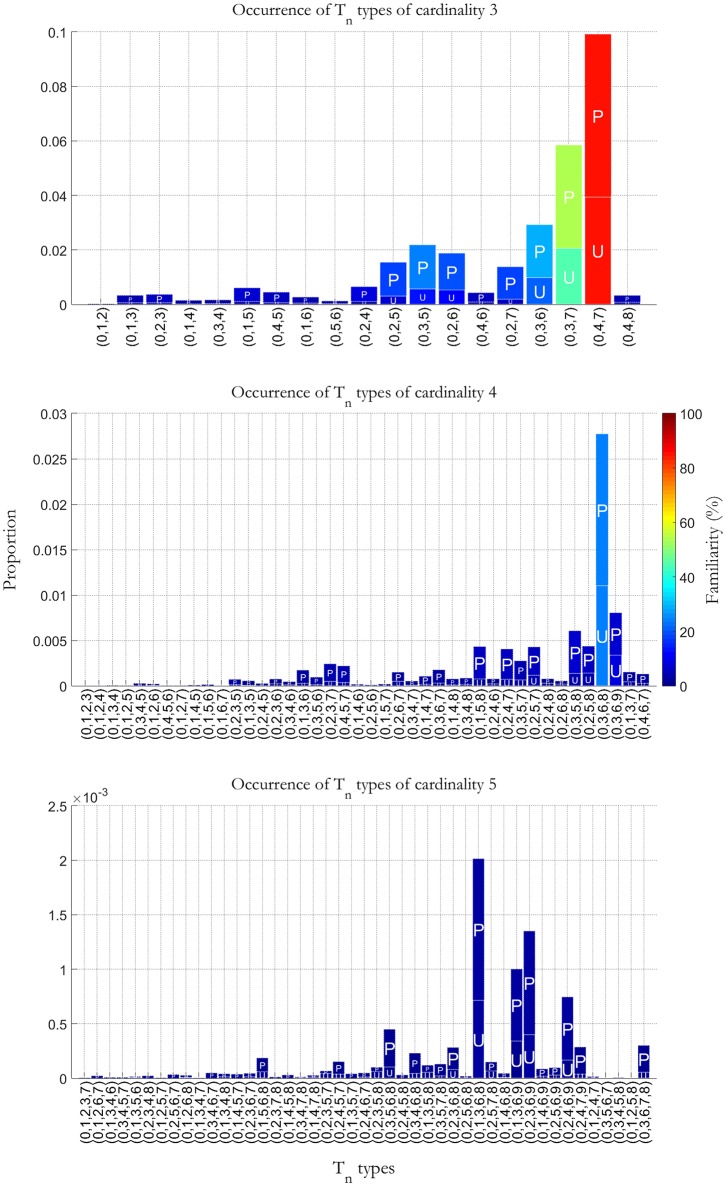
Occurrence of vertical T_*n*_ types of cardinality 3, 4, and 5 as a percentage of the total number of counted sonorities in a database of Western tonal piano music comprising the complete Bach's *Well Tempered Clavier*, a selection from the *Sonatas* by Domenio Scarlatti, all Beethoven piano *Sonatas* and *Variations*, all Chopin *Mazurkas* and *Preludes*, and a selection of pieces by Schumann and Brahms. *P* stands for prepared sonorities and *U* for unprepared sonorities. The color scale on the right side of the figure represents vertical T_*n*_ types' familiarity.

The three panels in Figure 2, respectively show the frequency of occurrence of vertical T_*n*_ types of cardinality 3, 4 and 5 (i.e., sonorities with simultaneous occurrences of 3, 4, and 5 sounds), as a percentage of the total number of counted sonorities in the selected database. For example, (047) in the upper panel corresponds to the major triad, which is also the most common chord sonority in the database. Other examples of sonorities with a high degree of familiarity are the minor triad (037) and the diminished triad (036). Some T_*n*_ types in this panel do not correspond to trichords (simultaneities of three pcs), but to incomplete tetrachords (4 pcs): for example, in most cases (035) is associated to a seventh chord where the third is missed. The other two panels of Figure 2 can be understood similarly. The higher the bars, the more a T_*n*_ types is assumed to be familiar. *P* stands for prepared sonorities and *U* for unprepared sonorities. The color scale on the right side of each figure represents vertical T_*n*_ types' familiarity.

Like all pc-set analyses, our analysis has a weakness, namely the neglect of octave register and hence of the inversion, spacing, and doubling in each sonority. This practical limitation is difficult to overcome, given the enormous number of possible chords that become available when register is included in a systematic approach. In spite of this limitation, the predictions correspond well with our intuitions.

In our algorithm, the salience for the vertical harmonic accent of a sonority *i* is defined as the product of two numbers: 1 minus the number of times *T*_*n*_(*h,k*) that the *k*th T_*n*_ type of cardinality *h* (*h* = 1, 12) appears in our database, and the duration *d*_*i*_ of such sonority *i*. Despite the assumption that the greater the chord duration, the more clearly its vertical harmonic accent is perceived, if a notated duration exceeds one bar length the salience of the corresponding accent would be overestimated. Therefore, notated durations are adjusted in the following way. Let *d*_*n*_ be the notated duration of a sonority as related to the beat (i.e., *d*_*n*_ = 1 for a notated duration of 1/4 in 3/4 or 4/4 meter, and *d*_*n*_ = 0.5 for a notated duration of 1/8 in 3/4 or 4/4 meter; *d*_*n*_ = 1 for a notated duration of 3/8 in 6/8 meter) and *n*_*b*_ the number of beats in a bar (i.e., *n*_*b*_ = 3 for a notated measure of 3/4). In order to have a uniform measure of *d*_*n*_ for pieces with different notated tempi, a rescaling factor that halves the durations in case of fast notated tempi (e.g., *Allegretto, Allegro, Vivace, Presto*) is introduced. The predicted scaled duration *d* of a sonority is then:

(5) $$\begin{array}{l} d\;=d_{n}\;for\;d_{n}\leq 1\\ d=d_{n}^{1/2}\;for\;1<\;d_{n}<n_{b}\\ d=n_{b}^{1/2}\;for\;d_{n}\;\geq \;n_{b} \end{array}$$

The predicted vertical accent *H*_*v,i*_ at sonority *i* is calculated according to the formula:

(6) $$\begin{array}{l} H_{v,i}={\left\{1-\frac{T_{n}\left(h_{i},k_{i}\right)}{\max\left[T_{n}\left(k\right)\right]}\right\}}^{P_{v}^{2}}\cdot S\cdot d_{i} \end{array}$$

where *P*_*v*_ is a second rescaling coefficient depending on the style (see Table 3 above).

**Table 3:** Parameter values for harmonic accent in Model 2.

**Musical style**	***P_***v***_***	***P_***h***_***	***x*_**1**_**	***x*_**2**_**	***x*_**3**_**
Baroque—Classical	2	2	5	0.6	1
Romantic	2	2	4	0.3	1
Late-Romantic	3	3	4	0.2	1.5

The intention of the style-dependent parameterization is not to draw a quantitative comparison between styles—a task that would require a larger dataset and further investigation—but to weight the contributions to harmonic accents relative to each other in a stylistically appropriate way. *S* = 5 rescales from the range [0; 1] to the range [0; 5], and the reason for squaring *P*_*v*_ is to get a more balanced ratio between extremely dissonant and extremely consonant T_*n*_ types.

#### Horizontal Harmonic Accents

Our algorithm to predict the positions and saliences of horizontal harmonic accents has two separate stages. First, a pitch-class salience profile *Pcsal*_*i*_—a vector with salience values for the 12 pitches in the chromatic scale—is calculated for each sonority *i* (considered as a T_*n*_ type) using the root-finding model of Parncutt (1988). Second, for each sonority we compare the pitch-salience profile *Pcsal*_*i*_, as calculated over the 12 pitch classes, with the profiles of each previous sonority within a window of one measure by means of Pearson's correlation coefficients:

(7) $$\begin{array}{l} r_{ij}=\text{corr }\left(Pcsal_{i}\;,Pcsal_{i-j}\right)\;,\;j=1,\;2,\;\ldots ,N \end{array}$$

where *N* is the number of note/chords inside the window.

The lower the correlation, the more that sonority is predicted to be harmonically surprising or tense. Each correlation coefficient is weighted relative to each other to account for memory decay—earlier profiles getting less weight and later (most recent) getting more weight. The weights *w*_*j*_ are equal to *1/j*, where *j* is the distance between sonorities without considering durations or IOIs (so that *j* = 1 for the immediately preceding sonority and *j* = 2 for the sonority before that). Negative correlations *r*_*ij*_ < 0 are replaced with 0. We also tested the model for horizontal accents in the case negative correlations were preserved, and then the range of correlations rescaled from [−1; 1] to [0; 1]. Both solutions introduce some degree of approximation in the final calculation, overestimating some accents and underestimating other ones. However, the results provided by the two algorithms are very close to each other.

The predicted horizontal accent ***H**_**h,i**_* at sonority *i* is calculated according to the formula:

(8) $$\begin{array}{l} H_{h,i}={\left[1-\frac{\sum_{j=1}^{N}r_{ij}\cdot w_{j}}{\sum_{j=1}^{N}w_{j}}\right]}^{P_{h}}\cdot S\cdot d_{i} \end{array}$$

where the duration *d*_*i*_ of the sonority is calculated according to Equation (5), *P*_*h*_ is a rescaling coefficient related to the style similar to *P*_*v*_ previously defined for vertical harmonic accents (see Table 3), and *S* = 5 is the same parameter as described above. In sonorities consisting of different note values, the notated duration *d*_*n*_ is taken as the longest notated duration. Two other rescaling factor are introduced in the following cases: (i) doubling the window length and the number of beats *n*_*b*_ in case of half time signature, and (ii) halving the notated durations *d*_*n*_ in case of pieces including many consonant passing tones whose notated durations are comparable with the duration of the beat. The reason in the latter case is that although these sonorities correspond to a root change, they do not carry harmonic accents. In the repertoire analyzed in this study, there is no piece featuring the first condition, and only one piece featuring the second one (i.e., excerpt No. 1 from Mozart Sonata K. 533, 2nd Mvt.).

#### Harmonic Accents

Harmonic accents are estimated at each sonority by combining the algorithms for vertical and horizontal harmonic accents in two ways. A first model (Model 1) is defined by selecting the maximum between the two components *H*_*v*_ and *H*_*h*_ (Equations 6, 8):

(9) $$\begin{array}{l} H_{1}=max\;\left(H_{v}\;,\;H_{h}\right) \end{array}$$

The advantage of this formulation is undoubtedly its simplicity. However, from music theory we know that vertical dissonance and root change are differently balanced across different styles (Piston, 1987). Therefore, in a second and more accurate model (Model 2), the following parameterization system is introduced to increase the relative importance of root change from Classic to Romantic and late-Romantic repertoires:

(10) $$\begin{array}{l} H_{2}=max\;\left(\;H_{v}+\frac{H_{h}}{x_{1}}\;,\;5\right)\;for\;H_{v}\leq x_{2}\\ H_{2}=max\;\left(H_{v}\;,\;H_{h}\right)\;for\;H_{v}>x_{2} \end{array}$$

Besides the power-law coefficients *P*_*v*_ and *P*_*h*_ introduced above for vertical and horizontal harmonic accents, this second model includes three new parameters: the first and second parameters, *x*_1_ and *x*_2_, account for the relative importance of the two typologies of harmonic accents; then, resulting *H*_2_ values smaller than a cut-off parameter *x*_3_ are set to 0 to neglect the smallest contributions as a matter of clarity, by analogy to our procedure for metrical and melodic accents. Values of the three parameters *x*_1_, *x*_2_, *x*_3_ are reported in Table 3. We manually adjusted the parameters by comparing predictions for a first group of 3 pieces with musical and music-theoretical intuitions and with the results of Exp. 2. We then considered predictions for the remaining 7 pieces. Model 2 was tested on another set of 20 pieces belonging to a different study (styles from Baroque to post-Romantic equally represented, 1,476 sonorities; not reported in this paper).

Model 2 works in the following way. First of all, we noticed that in our model a predicted harmonic vertical accent always corresponds to an intended accent, but a predicted horizontal accent (root change) does not. Let's see why. When dealing with passing/neighbor tones, passing/neighbor chords and suspensions, the model *H*_*v*_ or vertical accent (harmonic dissonance) works well because passing and neighbor tones have generally small durations and are therefore not accounted for, and passing, neighbor, and suspension chords are familiar in the database upon which the model of vertical accents is based (cf. Figure 2). On the other hand, the model *H*_*h*_ for horizontal accent (root change) cannot avoid wrong selections in the case of passing, neighbor and suspension chords with long durations, because they—although consonant or familiar and therefore not carrying dissonance—may produce root change although they are unaccented. When the harmonic dissonance is high, e.g., in the late-Romantic style, this problem is handled by Model 1. In fact, in this case either the harmonic dissonance is selected, or the horizontal accent is higher than the vertical accent, and this normally corresponds to situations when a root change really occurs. This is not the case when harmonic dissonance is low, e.g., in the Classical style. Here, Model 1, when selecting the maximum between *H*_*v*_ and *H*_*h*_, wrongly accounts for moderate root change as horizontal accents. Therefore, a new parameterization system is required to avoid this effect. In other words, we introduce a threshold (*x*_2_) above which we can still continue using Model 1, and below which we need to formulate a new model (Model 2), corresponding respectively to the lower and upper part of Equation 10. The threshold *x*_2_ determines the point where one changes from lower to higher harmonic complexity and is therefore depending on the style. In particular, *x*_2_ is higher for Baroque and Classical styles, and lower for Romantic and late-Romantic styles. Below the threshold (i.e., within the regime of Model 2), the overall harmonic accent is then modeled as a weighted sum of *H*_*v*_ and *H*_*h*_. Here, a contribution from *H*_*h*_ which increases with increasing harmonic complexity is added to *H*_*v*_ (as accounted by the inverse of the parameter *x*_1_).

A last case occurs when standard tonic-dominant chord progressions occur in late- or post-Romantic music. In this case, the model selected is the 2 (as harmonic dissonance is low), the threshold *x*_2_ is the lowest, and the weight 1/x_1_ for the horizontal harmonic model *H*_*h*_ is the highest (cf. Table 3). As a consequence, the model may predict some extra noise at the level of the threshold *x*_2_, and therefore the cut-off *x*_3_ is set as a little bit higher for the late-Romantic style. Finally, we apply a saturation rule assigning the value of 5 to all harmonic accents higher than 5 (a situation that may occur with Model 2), in the same way as for the metrical and melodic accents. These accents are pretty rare and their saliences just slightly higher than 5.

In this way, with a few number of parameters we reach three goals: (i) in the Classical style, reduce the relative importance of root change when it results from passing/neighbor tones/chords or unsurprising tonic-dominant progressions; (ii) in Romantic and late/post-Romantic music, reinforce the effect of root change to properly model modulations and surprise; (iii) when standard tonic-dominant chord progressions occur in late- or post-Romantic music, manage to treat them as in the earlier pieces. Note again that our purpose is not to model different styles, but to generalize our model across variations in harmonic complexity and musical style.

Note that in sonorities consisting of many notes of differing durations, the notated duration *d*_*n*_ is computed as the shortest notated duration in case of the vertical harmonic accents, and as the longest notated duration in case of horizontal harmonic accents. This correction has a psychoacoustical motivation: harmonic dissonance (i.e., vertical harmonic accents) may be conceived as a property of individual sonorities, while harmonic surprise (or horizontal harmonic accents) depends mainly on the harmonic function of sonorities—i.e., for example, passing and neighbor tones contribute to surprise less than structural notes/chords.

Figure 1 shows an example of how the harmonic accents are computed by the model. As we can see, harmonic accents are predicted on the first chord of bars 2 and 10, as they correspond to an increase of harmonic tension on the dominant. The same effect is observed in bars 5–6; here, the harmonic accent is less salient in the second repetition of the chord, but more salient in the third repetition when the chord duration is longer. Generally, the tonic has no tension (bars 3–4 and 7–8), unless it corresponds to a remarkable melodic movement (e.g., the melodic climax at bar 11, and the resolution to the tonic as a conclusion of the piece at bar 15). The double appoggiatura on the tonic at the start of measure 3 has horizontal tension, because its chroma-salience profile does not correlate strongly with preceding chords. From a music-theoretical perspective, this dissonance is resolved by stepwise (semitone) motion to the nearest consonance. The double appoggiaturas at the beginning of bars 11, 13 and 15 can be understood similarly. The tritones in measures 6–7 and 14 carry harmonic accents because they are dissonant and quite unfamiliar in our database. The chord at the start of measure 12 presents root change due to the movement on the secondary dominant (V^7^/ii), and therefore it carries an harmonic accent originating from harmonic surprise. The dominant ninth at the beginning of bar 14 has a very high harmonic accent, both because this chord is relatively unfamiliar in our database, and as the chord progression V^7^/ii–ii^87^–V^9^–I is quite surprising with respect to the rest of the piece and prepares the final cadence. This accent corresponds also to the harmonic climax of the piece. Other examples of accentuation are provided with the results of the experimental studies.

## Results

### Raters' Comments

In Experiment 1, the main result from the questionnaire was that some participants mentioned a lack of metrical/rhythmical accents in the musical examples. None of them reported that the task was difficult. In Experiment 2, raters' annotations consisted mainly of comments on the general feedback, points of criticism, reports on the difficulties encountered in the task, and suggestions for future improvement. In Experiment 2, two participants fully accepted our approach, although expressing some criticism. Another two offered suggestions for improvement, consisting mainly of new principles rather than changes to existing rules. In particular, it was suggested to include new categories of accents and to extend the current ones. New categories of accents that were proposed include: grouping/phrasing accents (at the start/end of phrases, i.e., after a pause or a cadence), textural accents (sudden textural changes, change of register, voicing, motivic grouping), thematic/motivic accents (accounting for repetitions of a previous theme/motive), dynamical accents (on single notes according to the dynamic indications in the score, at the beginning/climax of a crescendo or at sudden dynamical changes), articulatory accents (legato/staccato), and timbral accents (emphasizing octave doubling in the melodies, cantabile or spectral doubling).

Possible extensions of current categories of accents include, for metrical/rhythmic accents: syncopation or metric asynchronies/irregularities/instabilities, interruptions/reprises of rhythmic patterns, prolongation of up/downbeats, changes in the note durations, relationships with melodic accents or with other aspects not strictly related to the meter. Regarding melodic accents, participants suggested including directional inversions in the melodic progressions, connections by step of distant notes and/or non-conventional (i.e., diminished or augmanted) melodic leaps, and melodic passages carrying dissonances (passing or neighbor tones). For harmonic accents, some participants suggested explicitly including ideas from traditional tonal harmony theory—e.g., chords different from triads, harmonic tension to an expected sonority or climax, suspensions with resolutions, melodic/harmonic appoggiaturas, accidentals (especially with tensions toward the following note, or indicating a new key, or a relevant harmonic change not explicitly indicated in the score), harmonic pedal, harmonic sequences not corresponding to the ordinary fifth/fourth relationships (as in chromatic or neo-Riemannian shifts).

Although interesting and worth considering in later modeling, many of these suggested improvements did not easily fit with the requirement of a completely automatic model. As regards harmonic accents, most of the addressed issues are covered implicitly by the root change algorithm in our horizontal accent model.

Most participants complained that it was difficult to distinguish between separate categories or to choose a specific category of accents. They were also uncertain about selection of identical events. This feedback inspired us to extend the data analysis by combining different categories of accents, as discussed below (sections from Agreement Among the Raters to Overall Measure of Ratings). The participant with a Schenkerian orientation commented on the difficulty of relating his accent analysis to the voice leading or to associate temporally separate elements to each other. This participant also commented that, in a Schenkerian interpretation, accents do not correspond to “structural” events but rather coincide with linear sequences and/or melodic movements (*for me accents do always identify a movement, or a beginning of a movement, e.g., dissonances moving toward consonances)*. This analyzer also complained about the absence of expressive indications in the scores (however, contradicting his suggestion that expressive indications should be always predicted by music analysis). Other comments addressed the importance of listening, intuition, and analytic thinking (*I tried to be based only on what I was able to “listen,” without thinking too much; Only saliences that are perceivable by listening have been considered*), observations on the importance of distinguishing between real-time analysis and post-processing (*I required some time to adapt myself to the meter*), and interpretation of accents in terms of tension.

These comments opened an interesting window on a more accurate rationale of our approach, and are being in part addressed in a follow-up study (Friberg et al., submitted)^1^. To better understand the mental strategies of the participants, they might in a future study be asked to explain each annotation in a questionnaire or open interview.

### Overview of Accent Marks

The proportion of sonorities that were marked as accents in the different cases is reported in Table 4. Note that the percentages were rather consistent across each accent category, but with comparatively few harmonic accents for the Baroque/Classic category in Experiment 2 and comparatively more accents for the late-Romantic category.

**Table 4:** Proportion of sonorities that were marked as accents.

**Accent**	**Exp. 1**	**Exp. 2**		
	**Romantic (*n* = 239)**	**Baroque/Classic (*n* = 338)**	**Romantic (*n* = 260)**	**Late-Romantic (*n* = 40)**
Metrical		24.3%	35.4%	45.0%
Melodic	38.5%	28.7%	36.5%	55.0%
Harmonic	37.2%	16.9%	34.6%	45.0%

*n, number of participants*.

### A Qualitative Comparison Between Models and Data

**Figure 3:**
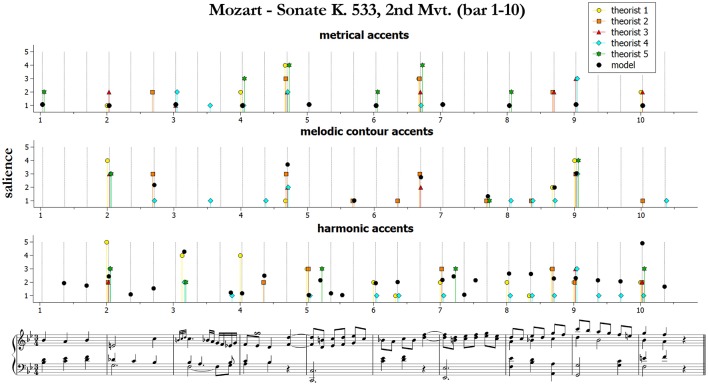
Accent marks and model predictions for Mozart Sonate K. 533, 2nd Mvt. (bar 1–10). The harmonic accents are those predicted by Model 2 (Equation 10).

**Figure 4:**
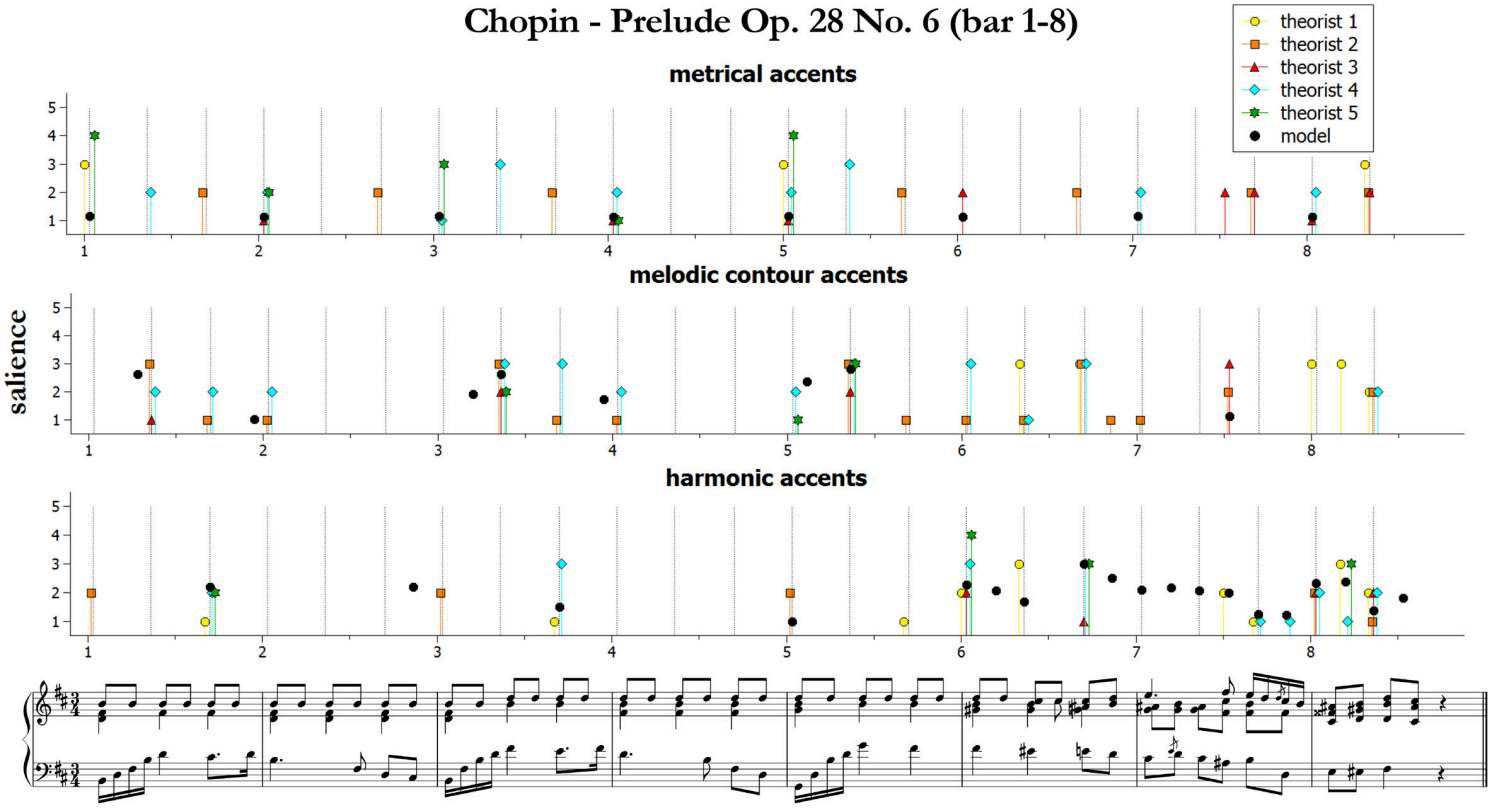
Accent marks and model predictions for Chopin Prelude Op. 28 No. 6 (bar 1–8). The harmonic accents are those predicted by Model 2 (Equation 10).

**Figure 5:**
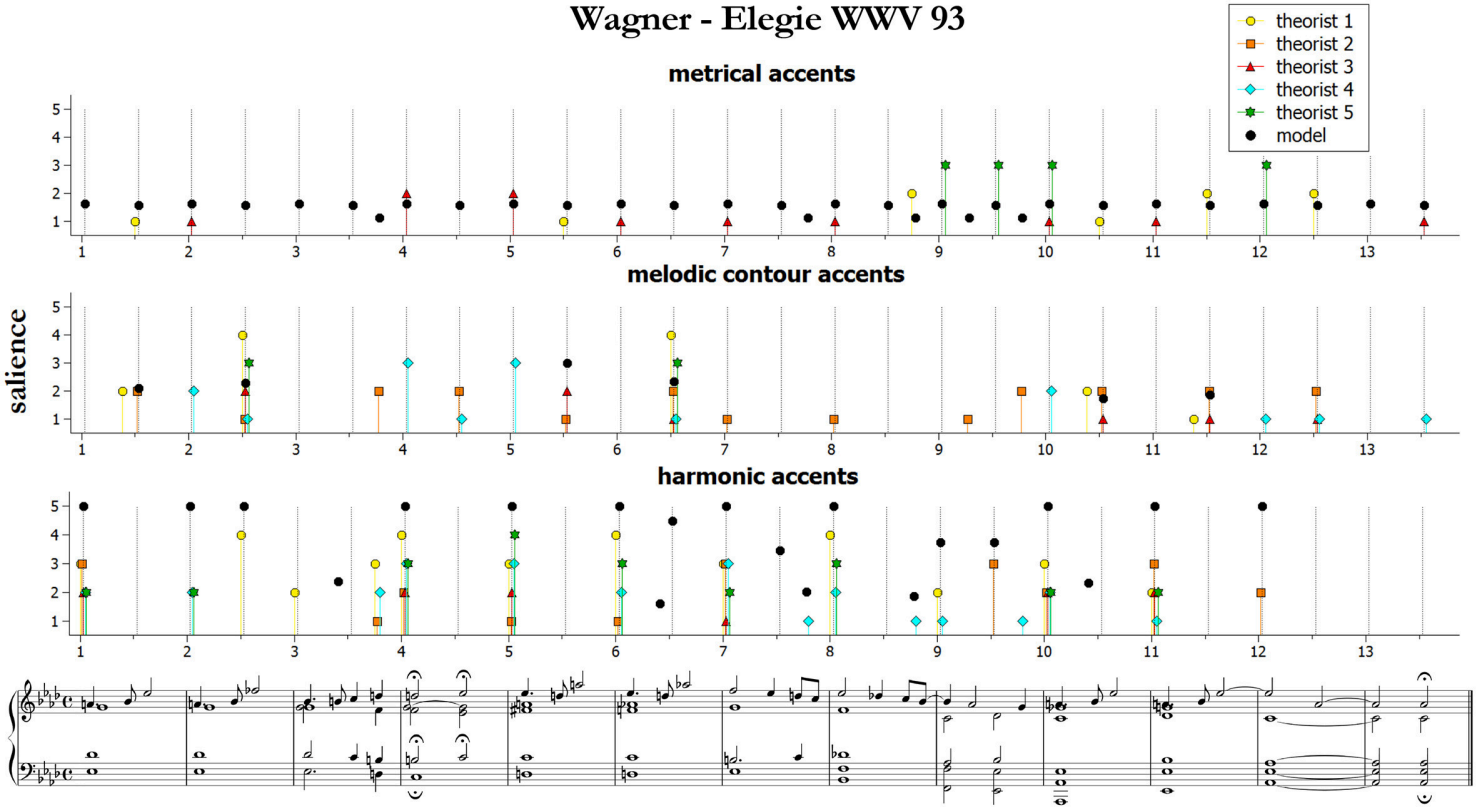
Accent marks and model predictions for Wagner Elegie WWV 93. The harmonic accents are those predicted by Model 2 (Equation 10).

We start by presenting and qualitatively discussing some examples^11^. Figures 3–5 illustrate ratings and model predictions from Experiment 2 for three examples, contrasting by musical structures and styles (cf. Table 1 above). Pieces are those indicated as excerpts n. 1, 6, and 10 in Table 1. As we already said, the limited number of participants did not allow us to establish any quantitative relationship between our model and their approaches to music analysis, therefore any comparison of this kind has to be treated as indicative. Apart for a small number of accents, the raters agree rather poorly with each other; however, their agreement is higher for events which received the highest ratings—see for instance the big melodic leaps at the beginning of bar 2, at the end of bar 4 and at the beginning of bar 9 of the Mozart excerpt (middle panel of Figure 3), the leaps on the 2nd beat of bars 1, 3, and 5 of the Chopin excerpt (Figure 4, middle panel), or the increasing harmonic tension in the chord progression I–IV#–V–I of bars 2–3, in the lower panel of Figure 3 (Mozart excerpt).

From the figures and the results from remaining pieces we can observe that of the three categories of accents, melodic contours and harmonic accents are those with better match between models and data. Conversely, theorists conceived metrical accents not only as related to the downbeats (as our model does), but also in strong relationship with melodic contour accents—see for instance the (not predicted) metrical and melodic contour accents on the 3rd beat of bars 4 and 6 of the Mozart excerpt (this effect is confirmed also by other pieces that are not shown here). Our model is sometimes too generous in modeling harmonic accents (as with the passing chords in the Mozart excerpt). The reason is that the model is strictly applied according to principles, while the raters often chose not to mark all the accents that would have been predicted. This is a subjective aspect difficult to be formulated into an automatic algorithm. Of the three pieces illustrated here, the post-Romantic Wagner excerpt of Figure 5 is the one featuring the strongest predicted harmonic accents, both vertical and horizontal; interestingly, this is also the case where the agreement is higher, both between the raters and between model and data.

From this qualitative inspection of the measurements, we noticed that when the agreement between the raters is higher, also the agreement between raters and model(s) is higher, in particular in Experiment 2. Therefore, we investigated whether the model, when treated as an additional rater, performed better that the average of all of the raters. In the following of this section, we will address these points quantitatively.

### Agreement Among the Raters

The agreement of the raters in terms of the individual variation was estimated by the average Pearson's correlation coefficient *r*^12^ between all pairs of raters across all music examples, see Table 5. *r* was calculated for both single and combined categories of accents (by selecting the maximum between two or three categories). All notes have been included and the ones without marks have been coded as zero. The total number of notes included in all of the pieces is 239 in Experiment 1 and 638 in Experiment 2. Thus, a value of 1 would represent a perfect match, while a negative value would indicate that most of the accents marks are on different positions. Note that, for separate categories of accents, correlations are comparatively lower than 0.50, which corresponds to a weak uphill relationship (Rumsey, 2010). This indicates that different raters presumably used different strategies for marking the accents. Interestingly, the agreement is somewhat higher for almost all categories of combined accents, indicating that raters might use also strategies differing from individual models' principles for evaluating pitch-time structures. Also note that the experts in Experiment 2 did not agree to a higher extent than the musicians in Experiment 1.

**Table 5:** Average pairwise correlation between all the raters.

**Accent**	**Exp. 1 (*n* = 16) (# of notes = 239)**	**Exp. 2 (*n* = 5) (# of notes = 638)**
M		0.27 (0.01–0.55)
C	0.41 (0.00–0.87)	0.33 (0.19–0.55)
H	0.48 (0.18–0.90)	0.50 (0.37–0.64)
MC		0.41 (0.27–0.58)
CH	0.51 (0.17–0.89)	0.49 (0.36–0.58)
MCH		0.57 (0.47–0.67)

*Total range is indicated in parenthesis. M, metrical accents; C, melodic contour accents; H, harmonic accents; MC, metrical-melodic accents; CH, melodic-harmonic accents; MCH, metrical-melodic-harmonic accents; n, number of participants*.

### Correlations Between Raters and Model

The average pair-wise correlation between each rater and corresponding models across all music examples are shown in Table 6. The combined categories, regarding both marked accents and models, are computed in the same way as before. In general, the agreement between raters and models is higher in Exp. 2 (experts in music theory) than in Exp. 1 (musicians). Interestingly, for Exp. 2 the values are rather close to the rater agreement values shown in Table 5, indicating that the model is on par with a typical expert rater. For the predictions involving harmonic accents, the result for both models are shown. Note that the agreement between raters and harmonic Model 1 is slightly higher than the agreement between raters and harmonic Model 2. This may be a consequence of the low agreement among the raters, who on average fit worse with a more accurate model.

**Table 6:** Average pair-wise correlation between models and raters.

**Model**	**Raters—Exp. 1 (*n* = 16) (# of notes = 239)**	**Raters—Exp. 2 (*n* = 5) (# of notes = 638)**
M		0.29 (−0.07–0.54)
C	0.39 (0.15–0.69)	0.32 (0.23–0.48)
H_1_	0.34 (0.08–0.53)	0.50 (0.43–0.62)
H_2_	0.32 (0.08–0.50)	0.45 (0.37–0.57)
MC	0.41 (0.19–0.65)	0.41 (0.35–0.52)
CH_1_	0.37 (0.15–0.54)	0.50 (0.47–0.59)
CH_2_	0.37 (0.16–0.53)	0.48 (0.45–0.53)
MCH_1_	0.37 (0.15–0.55)	0.54 (0.48–0.59)
MCH_2_	0.37 (0.16–0.54)	0.54 (0.49–0.57)

*Total range is indicated in parenthesis. M, metrical accents; C, melodic contour accents; H, harmonic accents; MC, metrical-melodic accents; CH, melodic-harmonic accents; MCH, metrical-melodic-harmonic accents; n, number of participants*.

We compared the correlation coefficients *r* between raters and models for metrical and melodic accents in the current formulation (separately and in combination) with those from the previous models (Friberg and Bisesi, 2014). For metrical accents, *r* was 0.25, i.e., the current model improves of 18%. For melodic accents, by computing the weighted average on the number of notes between the two experiments, we obtained *r* = 0.33 in the previous formulation and *r* = 0.34 in the current one. When combining metrical and melodic accents, we obtained *r* = 0.30 and 0.41, respectively (again, for the weighted average between the two experiments), i.e., our new formulation improves of the 24%.

### Overall Measure of Ratings

We combined all the raters into a single measure expressing the consensus of the raters in the following way. A note position was counted as an accent only if there were at least *m* raters who marked that note. The final accent for that note position was computed as the average across these remaining marked values. This new overall rating was then correlated with the different models in the same way as before. In the analysis, we varied the value of *m* for each model, and selected the values that gave the highest correlation with the models. The result is shown in Table 7. Note that the correlations for this overall rating measure are generally higher than the individual values in Tables 5, 6. This indicates that if the accents are selected on the positions where there is consensus among the raters, this constitutes a measure that is closer to the model. Interestingly, *m* = 8 for the melodic ratings and *m* = 4 for the metrical-melodic ratings in Exp. 1, while it is equal to 1, 2 or 3 in the other cases.

**Table 7:** Correlations *r* between the overall rating measures and the models.

**Model**	**Raters—Exp. 1 (*n* = 16) (# of notes = 239)**		**Raters—Exp. 2 (*n* = 5) (# of notes = 638)**	
	**Overall rating**		**Overall rating**	
	***r***	***m***	***r***	***m***
M			0.54	1
C	0.61	8	0.43	2
H_1_	0.57	2	0.62	1
H_2_	0.57	1, 2	0.61	1
MC	0.59	4	0.54	2
CH_1_	0.56	3	0.61	2
CH_2_	0.57	3	0.59	2
MCH_1_	0.57	2, 3	0.62	3
MCH_2_	0.58	2, 3	0.62	2

*M, metrical accents; C, melodic contour accents; H, harmonic accents; MC, metrical-melodic accents; CH, melodic-harmonic accents; MCH, metrical-melodic-harmonic accents; n, number of participants. A note position is counted as an accent only if there are at least m raters who marked that note. All correlations are significant at p < 0.001*.

Table 8 shows correlations between the overall combined models and rating measures, for all the 21 participants and the 2 pieces shared by the two studies (first 8 bars of Prelude Op. 28 No. 6, and Prelude Op. 28 No.7 by Fryderyk Chopin). As before, we combined all the raters into a single measure expressing their consensus, a note position is counted as an accent only if there are at least *m* raters who marked that note; then, the final accent for that note position is computed as the average across these remaining marked values, and we selected the *m* values giving the highest correlation with the models. In the combined models, accents correspond to the maximum between two or three categories of accents both for the models and the ratings.

**Table 8:** Correlations between all combined models and rating measures, for all the 21 participants and the two pieces in common with the two studies.

**Model (# of notes = 108)**	**Raters—Exp. 1 and 2 (*****n*** **= 21)**	
	**Overall rating**	
	***r***	***m***
MC	0.66	5
CH_1_	0.66	7
CH_2_	0.61	7
MCH_1_	0.66	2
MCH_2_	0.63	2

*MC, metrical-melodic accents; CH, melodic-harmonic accents; MCH, metrical-melodic-harmonic accents; n, number of participants. A note position is counted as an accent only if there are at least m raters who marked that note. All correlations are significant at p < 0.001*.

**Figure 6:**
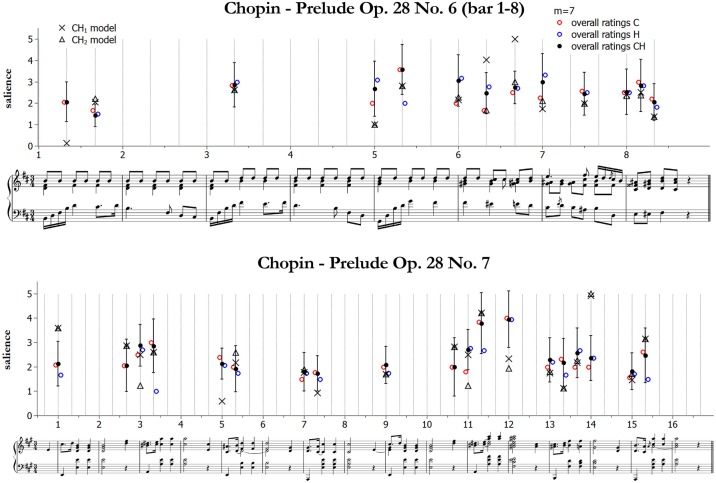
Comparison between all data (21 raters) and the models combining melodic and harmonic model accents, for the two pieces in common with the two experiments. Also shown are the separate contributions from the melodic (*C*, red/light open circles) and the harmonic model (*H*, blue/dark open circles). Error bars indicate standard deviations for the combined ratings (*CH*). **Upper panel:** first 8 bars of Chopin Prelude Op. 28 No. 6. **Lower panel:** Chopin Prelude Op. 28 No. 7.

Figure 6 illustrates an example of the resulting data from Table 8. Although not the one with the best fit, we chose to plot results for the less complex melodic-harmonic model as a matter of clarity. As we can see, there is an almost perfect agreement between models and ratings concerning the selection of notes. However, the accent values are different but are in a majority of cases (about 80%) close to each other and within the error bars of the rated values. Points of disagreement with respect to the melodic-harmonic model can be explained as follows. In Prelude 6, the marked melodic accent in bar 1 is modeled with low salience because its distance to the mean pitch is comparatively lower than for the other melodic peaks of the piece; the accent at the beginning of bar 5 is a melodic valley (carrying lower salience than melodic peaks, according to the melodic model) on a consonant chord, therefore there is only a small contribution to the melodic-harmonic accent, which is originated from the root change. In Prelude 7, the melodic accent in bar 1 has high salience because of the big leap with the preceding tone; the chord at the beginning of bar 5 is again a melodic valley on a consonant chord (low salience); the secondary dominant at the beginning of bar 12 has low salience because it is neither a melodic peak nor a dissonance, while the dominant ninth at the beginning of bar 14 has both dissonance (as it is less familiar in the database) and root change, and therefore it is very salient. Anyway, when considering the metrical-melodic-harmonic model most of these mismatches improve, as we can also infer from the better correlations reported in Table 8.

## Conclusions and Discussion

We have presented a computational model of accent salience that extends our previous formulation and is suitable for implementation into the existing rule system in Director Musices. The models of metrical and melodic salience are improvements to previous algorithms and a new automatic model for harmonic accents is introduced. The last one includes two typologies of harmonic accents: vertical dissonance and harmonic surprise in relationship to root change.

Our study includes two experiments involving intermediate-level musicians and expert music theorists, respectively. Participants marked metrical, melodic and harmonic accents on the score, and their strategies were commented in annotations. We define an agreement to be perfect if it corresponds to a correlation of 1, strong if larger than 0.7, moderate if comprised in the range 0.5–0.7, and weak if lower than 0.5 (Rumsey, 2010). In both experiments we obtained rather weak agreement both among the raters (corresponding to an average pair-wise correlation of 0.27–0.50, see Table 5) and between raters and model for individual metrical and melodic accents (average pair-wise correlation of 0.29–0.39, see Table 6); the agreement between raters and model for harmonic accents was a little bit higher (average pair-wise correlation of 0.32–0.50, see Table 6). For expert musicians (i.e., in Experiment 2), the agreement between different raters is comparatively consistent with that between raters and model, indicating that the model is on par with a typical expert rater. On this basis, we decided to combine all the raters into a single measure expressing their consensus, and this new overall rating was then compared with the different models in the same way as before. In this case, correlations with all of the models are higher than for individual values (average pair-wise correlation of 0.43–0.62; Table 7), indicating that—if handled as an additional rater—our model correlates with data better than how different raters do correlate with each other.

Besides the main task, comments by music theorists involved in Experiment 2 were also considered with the purpose of exploring their strategies for marking the accents in relation to mainstream approaches to music analysis. Two participants fully accepted our approach, while another two offered suggestions for improvement, consisting mainly of new principles rather than changes to existing rules. Interestingly, two out of the three theorists who agreed less with our approach were also the ones who performed “better” in terms of agreement with the models and with the other raters. The most significant outlier was the Schenkerian-oriented analyzer, who obtained the lowest match with both the models and the other raters. This is not surprising, as the Schenkerian approach differs substantially from accent-based approachs as in the current study. It aims at extracting the underlying structure of a piece in order to show how the surface of the piece relates to this structure; instead, our model is performance oriented and then focuses on tension and surprise at the level of the musical surface. Nevertheless, his suggestions were interesting in the future perspective of juxtaposing our model with traditional approaches to music analysis.

Despite reluctance by most of the participants to accept the reductionistic approach to separate the accents into three different categories, such prejudice did not seem to affect their capability to perform the tasks. However, following their reported disagreement with a reductionistic approach, a new data analysis was carried on to explore this effect. We combined different categories of accents into new categories involving two and three typologies of accent, by replacing values inside single categories with the maximum between all the categories. Surprisingly, in most cases correlations improved for combined ratings and models (see respectively Tables 5–7), confirming that raters might use strategies, such as interaction effects, different from individual metrical, melodic or harmonic accent formulations to mark musical events. As correlations' improvement for combined models is higher for single measurements than for overall measurements (shown in Table 7), low agreement between data and models might be related more to lack of consensus between participants (Table 5) than to the unreliability of the model. As consensus between participants increases, by complying participants' suggestion to combine different categories of accents into single ones or to make a selection of answers as in the overall measure (Table 7), the fit between model and data also improves.

Comparison between the two studies indicates higher agreement for melodic accents in Experiment 1, and higher agreement for harmonic accents in Experiment 2 (both among raters and between raters and models). This result might be interpreted as an effect of expertise: while melodic accents are quite intuitive and easily associated with everyday listening and/or performance at an intermediate level, harmonic accents are more complex and require deeper knowledge of music theory and analysis. However, due also to differences in the musical material, further studies are needed for confirming this conclusion.

Finally, we performed a further analysis concerning overall measure of ratings (again combining all raters into a single measure expressing their consensus) for all of the 21 raters and the two pieces in common with the two studies. Since combined models produced stronger results, we chose to look more closely at those, see Table 8. In this case and for this musical material, there is an almost perfect agreement between models and ratings concerning the selection of notes (Figure 6), and the accent values are in the majority of cases close to each other (average pair-wise correlation of 0.61–0.66, Table 8).

The current top-down accent formulation has some important strengths. First, when applied to automatic performance rendering, such an approach works more intuitively and is easier to compare with other methods for music analysis since the three components (rhythmic, melodic and harmonic) are separatedly modeled. Second, the local-context formulation of the model enables also to account for real-time perception of the musical foreground—an aspect of the greatest importance in expressive music performance. A third benefit concerns the possibility of introducing new methods for music education. Musicians learn about expression intuitively, imitate the expressive strategies of teachers and other performers, and gradually develop a personal voice. High-level performance teachers do speak analytically about the isolated ingredients of expressive performance, such as slowing down in particular ways at particular points or changing articulation to emphasize given events. However, they do so surprisingly rarely; many prefer to speak in impressive-sounding metaphors and images. That is perhaps because much of the relevant research is quite recent, and expression is associated more with intuitive rather than logical thinking. The analytical approach we have presented so far may help music students who are already inclined to think analytically about their practice to achieve their musical goals more quickly and easily, by stimulating their metacognitive ability to reflect on and develop their expressive strategies. In advanced music curricula, our approach could be linked to teaching of music theory and analysis, allowing students not only to analyze the pieces they are playing themselves (which motivates learning), but also how they are playing them.

In a follow-on project, the current top-down algorithmic formulation inspired by theoretical principles is being compared with a bottom-up model of immanent accent salience where a machine learning approach is used to extract a rather large set of features from the score representation and to express different hypotheses about perceptual accents, which include also the participants' suggestions on possible new categories of accents provided from the present study (Friberg et al., submitted)^1^.

Our model is based on widely accepted ideas in music theory, and its predictions are intuitively reasonable. Although further empirical work is needed to test whether this is a good foundation for performance rendering and, if so, how specific kinds of accents should be realized, this is a promising milestone. The task as a whole is challenging and multifaceted. First, it involves bringing together four disciplines with considerable epistemological differences: music theory/analysis, music information sciences, music psychology, and music performance. Second, it involves a complex system with several interacting but also partly independent variables. It is still possible that contrasting approaches to theory and modeling may produce predictions that are quantitatively equally good, and in that case other criteria will need to be found to evaluate the validity of the theories. However, having a model that is somehow competitive with mainstream approaches to music analysis, in that it looks at the musical foreground instead of background, is a situation that is much closer to performers' intentions and control.

## Ethics Statement

We believe that an ethics review process is not needed for our study. The task in which the participants were involved was an anonymous, written exercise. In Experiment 1 the participants were mostly adult music students (with the exception of two adult researchers in musicology) doing a music theoretic task. In Experiment 2 the participants were adult researchers in music theory, thus, doing an analysis within their research topic. Our studies did not involve any medical treatment and were carried out in accordance with the Declaration of Helsinki. All the data were totally anonymised, all subjects participated voluntarily in the study, gave written informed consent at the beginning of the task, and agreed to publish the results.

## Author Contributions

EB and RP conceived the original idea. EB and AF contributed to the conception and design of the study. EB, AF, and RP developed the metrical and the melodic model. EB developed the harmonic model (following also some suggestions by RP). EB performed the listening experiments. EB and AF performed the statistical analysis. EB wrote the first draft of the paper. AF contributed to the paper writing and editing. RP contributed to the Introduction and provided advice in English academic writing. All authors contributed to manuscript revision, read, and approved the submitted version.

### Conflict of Interest Statement

The authors declare that the research was conducted in the absence of any commercial or financial relationships that could be construed as a potential conflict of interest.

## Footnotes

^1^Friberg, A., Bisesi, E., Addessi, A. R., and Baroni, M. (submitted to Frontiers in Psychology - Section Performance Science). Probing the underlying principles of perceived immanent accents using a modeling approach.

^2^www.speech.kth.se/music/performance/download

^3^http://www.speech.kth.se/music/performance/download/dm-download.html, as implemented in Director Musices 3 Multiplatform (version 3.1.3).

^4^For a list of the structures involved in each piece, see Table 1.

^5^https://musescore.com/

^6^F. SCHUBERT – Impromptu Op. 142 No. 3, Theme.

^7^The time signatures provided in the table are only the most common ones and can easily be extended.

^8^We are assuming in a first approximation that the most salient pulses have a period of about two seconds (and not 600 ms as assumed by several studies), consistent with the idea that hypermeter affects timing and dynamics, and musicians tap slower than non-musicians when asked to tap the underlying beat of a piece of music (Drake et al., 2000).

^9^http://extras.humdrum.org/man/tntype/

^10^http://kern.ccarh.org/

^11^Data from Experiment 1 and 2 are provided as Supplementary Material.

^12^This is a very well-known quantitative method that has been used extensively in the past for evaluating participants' consistency and for comparing models with human annotations.
